# Plastic in seafood: are crustaceans a gateway to microplastic exposure in humans?

**DOI:** 10.1016/j.soh.2025.100121

**Published:** 2025-08-26

**Authors:** Mohammad Shakil Khan, Thowai Uching Marma, Samson Nahar Sumi, Aniruddha Chisim, Ifthekher Ahmed Shakib, Saifuddin Rana

**Affiliations:** a Department of Fisheries Resource Management, Faculty of Fisheries, Chattogram Veterinary and Animal Sciences University, Chattogram, Bangladesh; b Faculty of Fisheries, Chattogram Veterinary and Animal Sciences University, Chattogram, Bangladesh

**Keywords:** Microplastic pollution, Crustaceans, Ecotoxicology, Bioaccumulation, Seafood contamination, Human health risk

## Abstract

Crustaceans, widely consumed and ecologically significant marine organisms, are increasingly affected by microplastic (MP) pollution, one of the most pressing environmental challenges of the 21st century. These benthic and pelagic species, including shrimp, crabs, and lobsters, play essential roles in nutrient cycling, food web dynamics, and global seafood supply. The proliferation of plastic waste has led to widespread MP contamination in marine environments, threatening both ecological stability and human health. This review provides an in-depth overview of MP pollution, its ingestion and accumulation in crustaceans, and the resulting biological and toxicological effects. Data were compiled from leading academic databases, including Scopus, Web of Science, PubMed, ScienceDirect, and Google Scholar, with 54 peer-reviewed articles selected for synthesis. Fibers, fragments, films, and beads were the most frequently reported MP types, predominantly found in the digestive tracts, gills, and hepatopancreas of decapod crustaceans. These particles can cause oxidative stress, inflammation, reproductive disruption, and immune system impairment in crustaceans, while also acting as vectors for hazardous chemicals such as heavy metals and endocrine-disrupting compounds. The consumption of contaminated crustaceans poses potential health risks to humans, including gastrointestinal disorders, hormonal imbalances, and carcinogenic effects. Despite rapid progress in this field, major gaps remain in understanding the long-term ecological and human health impacts, particularly in less-studied regions and species. Further global investigations, long-term ecological assessments, and public awareness initiatives are essential. Effective mitigation will require interdisciplinary collaboration, technological innovation, and sustainable waste management to ensure a healthier marine ecosystem and safer seafood consumption.

## 1 Introduction

### 1.1 Microplastic (MP) pollution: sources and environmental presence

**Fig. 1:**
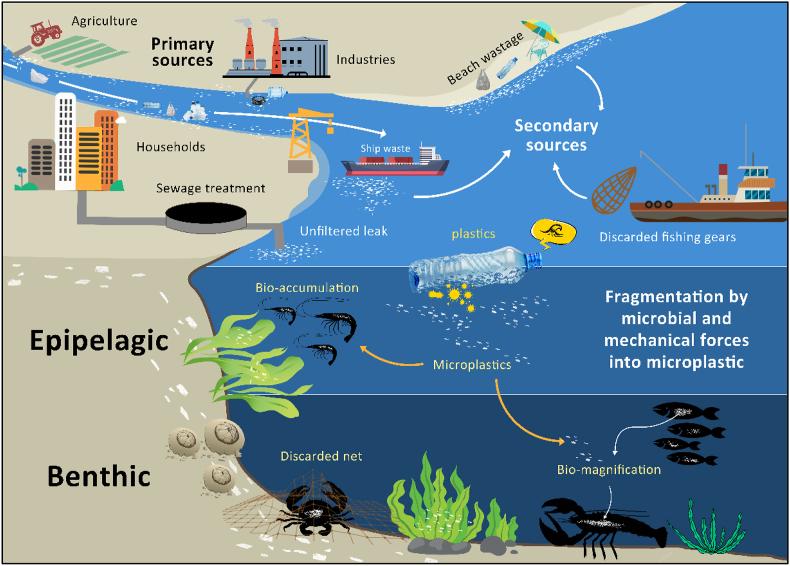
The sources, fragmentation, and bioaccumulation of microplastics in marine ecosystems and their effects on crustaceans.

MP pollution has emerged as a pervasive environmental challenge of the 21st century [1]. MPs are plastic particles smaller than 5 mm, originating from the fragmentation of larger plastic debris or manufactured as microbeads and fibers used in consumer products [2,3]. Common sources include synthetic textiles, tire abrasion, landfill leachate, sewage sludge, and inadequate waste management practices [4]. Fig. 1 illustrates the main sources and fragmentation pathways of microplastics entering aquatic ecosystems, setting the stage for their subsequent accumulation in marine organisms. Due to their small size, chemical stability, and persistence, MPs accumulate extensively in aquatic environments such as oceans, rivers, and freshwater systems [5]. These particles are typically categorized as fibers, fragments, films, or beads, each with distinct physical and chemical properties influencing their environmental fate and biological interactions [6].

MPs pose substantial risks as they serve as vectors for hazardous chemicals, including heavy metals and persistent organic pollutants [7,8]. Their widespread presence disrupts aquatic ecosystems by infiltrating food webs [9] and impacting organism health at multiple trophic levels [10,11]. Coastal regions and areas near urban and industrial centers experience especially high MP contamination due to concentrated anthropogenic activities and waste discharge [12].

### 1.2 Ecological and economic importance of crustaceans

Decapod crustaceans such as crabs, shrimp, and lobsters are integral to marine and freshwater ecosystems. They contribute to nutrient cycling, sediment bioturbation, and serve as both predators and prey within complex food webs [13,14]. Predatory crustaceans can help control the populations of other organisms, contributing to the overall health and balance of the ecosystem [15]. Crustaceans also hold considerable economic value as crabs, shrimps, lobsters, and prawns are a globally important food source for humans [16]. They are a significant component of both wild fisheries and aquaculture industries, providing livelihoods and contributing to economic development [17].

The feeding habits of crustaceans, which include filter feeding, scavenging, and detritus consumption, expose them to significant MP contamination [18]. MPs can be ingested directly or adhere to their exoskeletons, facilitating uptake into their tissues and potentially affecting their health and ecological functions [19]. Recent studies have shown that a large proportion of marine crustaceans contain MPs, which indicates their susceptibility to contamination. This accumulation suggests the potential for trophic transfer of MPs within marine food webs, impacting not only the organisms themselves but also higher trophic levels, including fish and humans [20,21]. MP pollution in aquaculture areas can disrupt the crustacean supply chain, as it may impair growth, reproduction, and immune function in commercial species [22]. Although the bioaccumulation of MPs in crustaceans is generally low, prolonged exposure can affect the overall health of aquaculture species, potentially reducing productivity and impacting seafood supply.

### 1.3 MP uptake in crustaceans

MPs enter crustacean bodies primarily through feeding on contaminated sediments, plankton, or other food items [23]. MPs can enter the body through the gills, where they are filtered from the water [24]. To a lesser extent, it can also enter via attachment to external surfaces such as exoskeletons. These particles have been detected in various tissues, including gastrointestinal tracts, gills, and hepatopancreas [25] (Fig. 1), with smaller MPs (<1 mm) tending to accumulate more readily and exert greater toxicological effects [18,26]. Species-specific feeding strategies, habitat types, and environmental contamination levels strongly influence MP uptake and accumulation patterns [27,28]. Polymer types commonly found in crustaceans include polyolefin, polyester, and polyamide, particularly in fibrous form [29].

### 1.4 Ecological implications of MP contamination

**Fig. 2:**
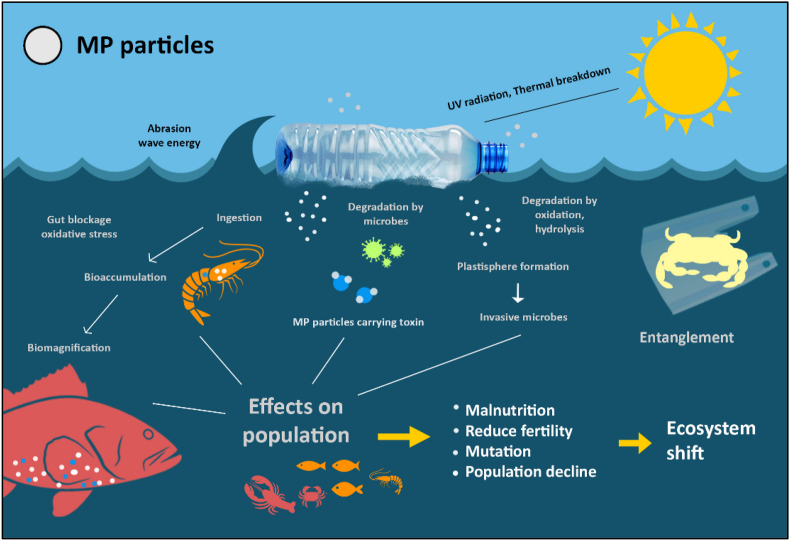
Ecological pathways and consequences of microplastic contamination in marine organisms.

MP pollution can affect various levels of the marine food web, impacting plankton, invertebrates, fish, and marine mammals, potentially leading to population declines and shifts in ecosystem structure [30]. MPs can be ingested by marine organisms, from plankton to large fish and mammals, causing internal injuries, digestive issues, and reduced feeding efficiency (Fig. 2) [31]. In aquatic environments, MPs inhibit microbial activity and harm zooplankton and benthic organisms by obstructing digestion, leading to malnutrition and mortality [32]. Moreover, Larger pieces of plastic debris, including those that break down into MPs, can entangle marine animals, leading to injury, suffocation, or drowning [7]. Their ability to bioaccumulate through trophic transfer threatens higher predators and facilitates the transport of toxic pollutants, pathogens, and invasive species. These effects collectively reduce biodiversity, impair ecosystem functions, and pose potential risks to human health via contaminated seafood consumption [33].

### 1.5 Significance of the study

MP pollution in marine ecosystems is a growing global concern. However, significant gaps remain in understanding the long-term ecological and human health impacts of MPs, especially with respect to less-studied regions, species, and pathways of exposure. While previous studies have examined MPs in marine organisms, much of the literature is fragmented, focusing on short-term exposures or individual species, often excluding crustaceans despite their ecological and dietary importance. In particular, species-specific bioaccumulation patterns, tissue-level distribution (e.g., edible tissues), and the potential for trophic transfer to humans via seafood consumption remain underexplored. These unresolved questions hinder our ability to evaluate the full extent of MP risks in seafood safety. This review takes a novel integrative approach by synthesizing data across crustacean species to analyze not only the ecological roles and physiological responses of decapods to MP exposure but also the pathways through which MPs may enter human food chains. Special attention is given to edible tissues, geographic inclusion of understudied regions, trophic transfer mechanisms, and areas that remain insufficiently studied. By connecting crustacean ecology, MP pathways, and human health risks, this review offers a comprehensive foundation for public health risk assessments, regulatory frameworks, and future research priorities.

## 2 Materials and methods

### 2.1 Study search design and selection criteria

This review employed a systematic and thorough literature search approach to investigate and integrate the current understanding of MP contamination in decapod crustaceans. To structure the review design, the Preferred Reporting Items for Systematic Reviews and Meta-Analysis (PRISMA) guidelines were followed, ensuring methodological transparency, reproducibility, and comprehensiveness in data collection and synthesis.

In the execution phase, a systematic search was performed across various esteemed academic databases, such as Scopus, Web of Science, ScienceDirect, PubMed, and Google Scholar, to ensure the incorporation of reliable and high-quality sources. The aim was to gather the latest, relevant, and scientifically valid studies that enhance the understanding of the occurrence, effects, and consequences of MP ingestion and accumulation in decapod species.

To operationalize the search, strict inclusion and exclusion criteria were applied. Studies were excluded if they did not focus on MP contamination in decapod crustaceans (crabs, lobsters, and shrimp) or did not address the biological or ecological impacts of MPs. Topic relevance was assessed by reviewing abstracts and titles to ensure the study specifically examined MPs in the species of interest, excluding those focused on unrelated environmental pollutants or non-crustacean species. Emphasis was put on peer-reviewed journal articles, including both original research and review papers, that focused on MP contamination in marine decapods. The literature search was limited to English-language studies to ensure consistency and clarity in data interpretation. While this may exclude relevant non-English studies, it was necessary to maintain a manageable scope and ensure the quality of the included research. The literature search was also conducted without temporal constraints, recognizing the significance of both fundamental research and recent advances in the discipline.

This structured methodology supported the synthesis stage by enabling a comprehensive understanding of the development and present condition of studies regarding MP contamination in decapod crustaceans. The methodology facilitated a complete and well-informed summary, highlighting the environmental and possible health risks to humans related to MP exposure in this important group of water-dwelling animals.

### 2.2 Search strategy and keyword formulation

**Fig. 3:**
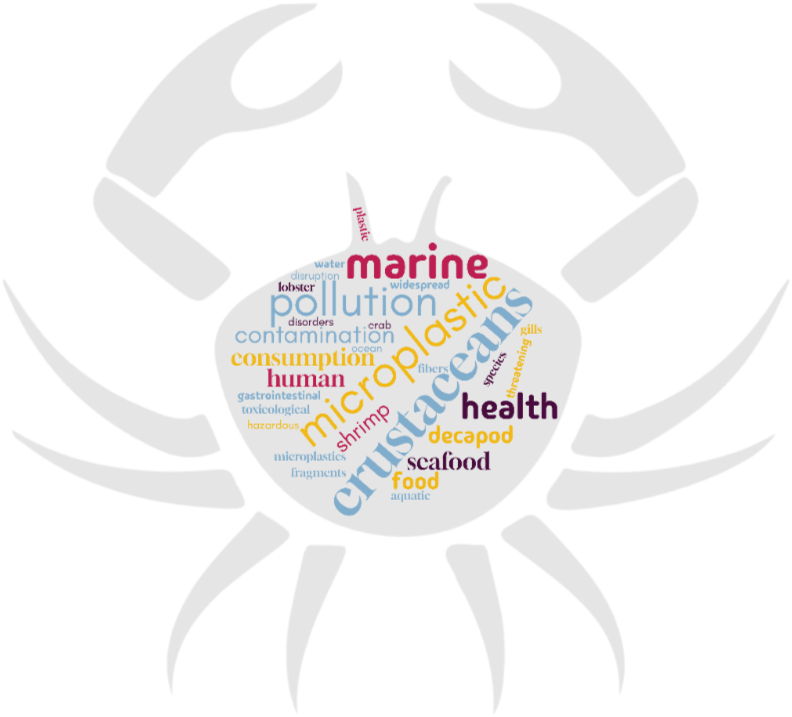
A word cloud was generated with the titles, abstracts, and keywords from the analyzed papers at https://www.wordclouds.com/.

A meticulously designed keyword strategy was applied to ensure a targeted and thorough collection of pertinent literature. A strategic selection of specific search terms related to MPs and decapod crustaceans was employed across chosen databases. Terms including “microplastics,” “decapod crustaceans,” “microplastic in shrimp,” “microplastic in crab,” “microplastic in lobster,” “microplastic contamination in crustaceans,” “microplastic pollution in the world,” “microplastic contamination in aquatic animals,” “effect of microplastic pollution on human health,” and “microplastic pollution in water” were frequently employed alongside Boolean operators such as AND and OR to enhance or narrow search outcomes based on relevance (Fig. 3). Quotation marks and truncation symbols were utilized as necessary to reflect variations in terminology while maintaining the contextual integrity of the search.

The keyword combinations were chosen to ensure a thorough exploration of the topic while specifically focusing on MP contamination in decapod taxa. These criteria supported the incorporation of a wide range of studies that examined various geographic areas, species, and environmental contexts. The adaptable search syntax enabled real-time modifications of queries to more effectively meet the study goals, thus improving the acquisition of both fundamental and innovative research. This organized method was essential for making sure that the final selection of literature was relevant and thorough, which helped create a solid understanding of MP exposure in decapod crustaceans and its broader ecological effects.

### 2.3 Data screening and selection process

**Fig. 4:**
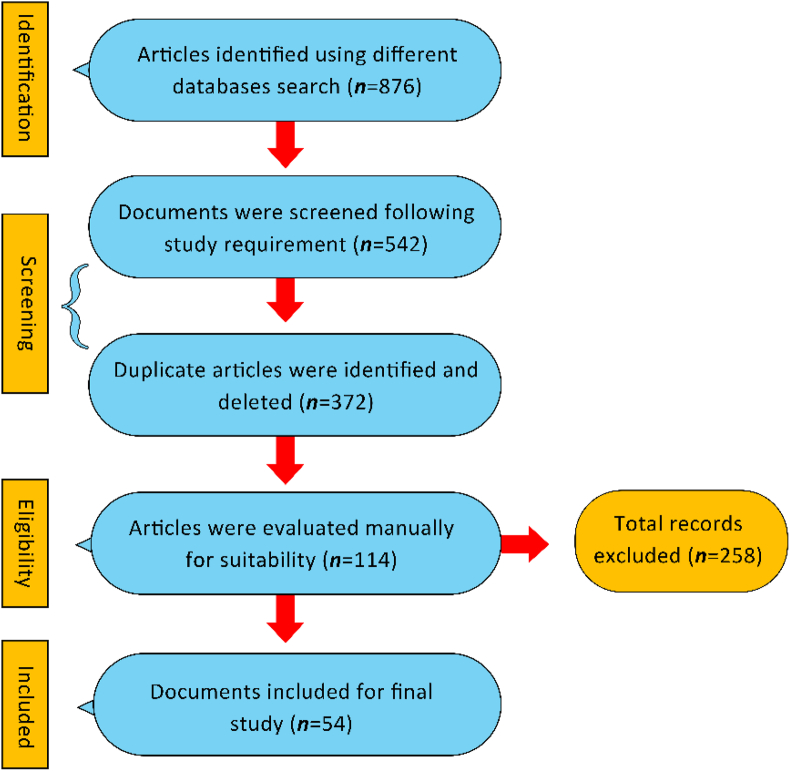
Process for searching and screening data records according to Preferred Reporting Items for Systematic Reviews and Meta-Analysis (PRISMA) guidelines.

A total of 876 publications concerning MP contamination in decapod crustaceans were identified in the initial database search. Following the application of the predefined inclusion criteria, which emphasized peer-reviewed articles, topic relevance, and publications in English, the total was narrowed down to 542 records. Identifying and eliminating duplicate entries across various databases led to a finding of 370 unique articles. The records underwent a thorough manual screening process, in which titles, abstracts, and when required, full texts were examined for their consistency with the research objectives. Subsequent to this evaluation, a total of 114 articles were selected for comprehensive full-text assessment. A total of 54 articles were identified as highly relevant and subsequently chosen for qualitative synthesis and data extraction. The process of screening and selection was meticulously documented in a systematic and transparent way, adhering to the PRISMA framework. Fig. 4 presents a flow diagram that outlines the stages of identification, screening, eligibility assessment, and final inclusion of studies, offering a visual summary of the bibliometric refinement process. This thorough multi-stage process guaranteed that the final collection of literature was both scientifically sound and closely aligned with the objectives of the review.

## 3 MPs in crustaceans, impacts, and mitigation approaches

### 3.1 Ingestion of MPs by crustaceans

Crustaceans are exposed to MPs by different pathways, mainly through their food sources, water, and sediment [26,27]. Crustaceans, including crabs and lobsters, can unintentionally take in MPs through the consumption of prey or detritus that is contaminated [34]. Deep-sea crustaceans have been observed to consume MPs, with differences in ingestion rates associated with the unique feeding strategies of various species and their environmental exposure [35,36]. The particles might also attach to their exoskeletons, potentially being consumed during molting or feeding activities [37]. Upon entering the gastrointestinal tract, MPs can either transit through or be assimilated into various tissues, including the gills, hepatopancreas, and intestines [37].

Various factors affect the uptake and accumulation of MPs in crustaceans, such as the characteristics of the particles, the surrounding environmental conditions, and the specific traits of different species. Smaller MPs, measuring less than 1 mm, are more likely to be consumed and result in more significant biological impacts than their larger counterparts [18]. Environmental factors such as habitat type, feeding behavior, and proximity to anthropogenic activity also significantly influence MP uptake in crustaceans [5]. Moreover, aquaculture practices, particularly in shrimp farming, can exacerbate MP exposure due to the use of plastic-based materials in feeds and farming gear [38]. Several studies emphasize that crustaceans from regions with high coastal plastic pollution exhibit higher MP loads, underscoring the influence of local environmental conditions [39]. Fibrous MPs are frequently identified in crustaceans [40], indicating that these forms may be more abundant in marine ecosystems or are selectively consumed by these species. The polymer type plays a significant role in uptake, with polyester and polyolefin frequently found in crustaceans [41]. These polymers are commonly present in fibers, representing the most frequently consumed type of MPs. Crustaceans exhibiting diverse feeding strategies demonstrate distinct levels of MP ingestion. Deposit-feeding crabs exhibit greater MP burdens in comparison to herbivorous or omnivorous species [41]. The consumption of marine snow and gelatinous substances is associated with higher ingestion rates [27]. The abundance of MPs in crustaceans also can be influenced by factors such as proximity to land and local environmental conditions, including fishing intensity and water velocity [42]. The influence of species-specific traits on MP uptake is significant. It has been observed that smaller crabs and those with shorter lifespans tend to accumulate higher levels of MPs [27]. Furthermore, non-migratory species typically exhibit greater ingestion rates than their migratory counterparts [28]. The consumption of MPs can impact the health of crustaceans and may facilitate the transfer of contaminants through the food chain, potentially affecting humans who eat them.

### 3.2 Occurrence of MPs in crustaceans

Decapod crustaceans play a crucial role in both ecological and economic contexts, constituting a vital part of global fisheries and marine food webs [17,43]. Their extensive consumption, especially in coastal areas, raises considerable concerns about MP contamination. Research has demonstrated the occurrence of MPs in the digestive systems, gills, hepatopancreas, and muscle tissues of different decapod crustaceans [37,44,45]. For example, MPs were found in the gastrointestinal tracts of key shrimp species, such as *Penaeus monodon*, *Penaeus indicus*, *Metapenaeus dobsoni*, *Penaeus merguiensis*, and *Metapenaeus monoceros*, with fibers and fragments being the most frequently identified types of polymers [46]. A study conducted by Hossain et al. [47] identified a total of 33 and 39 MP items in tiger shrimp (*P*. *monodon*) and brown shrimp (*M*. *monocerous*), respectively, with averages of (3.40 ± 1.23) items/g and (3.87 ± 1.05) items/g found in the gastrointestinal tract, respectively, within the waters of the Northern Bay of Bengal.

In crabs such as *Portunus sanguinolentus*, *Carcinus maenas*, *Neohelice granulate*, *Scylla serrata*, *Portunus pelagicus*, *Portunus trituberculatus*, *Charybdis japonica*, *Dorippe japonica*, and *Matuta planipes*, MPs have been noted to accumulate in the gills and hepatopancreas [[48], [49], [50], [51], [52]]. A study conducted by McGoran et al. [53] revealed that 71.3 % of the native shore crab (*C*. *maenas*) and 100 % of the Chinese mitten crab (*Eriocheir sinensis*) had at least one item (fibre, film, fragment, or tangle of fibres) present in their gill chamber and gastrointestinal tract.

Lobsters, as benthic and scavenging organisms, exhibit a notable vulnerability to the ingestion of MPs found within sediments and organic matter [54]. A study by Mutić et al. [20] revealed that 43.68 % of *Nephrops norvegicus* (langoustine or Norway lobster) samples were found to contain MPs, averaging less than one MP particle per individual across 190 samples. A separate investigation revealed that 85 % of the Norway lobster specimens analyzed contained a greater quantity of MPs in their guts compared to their stomachs, measuring (0.23 ± 0.16) mm and (1.00 ± 0.16) mm, respectively [55]. Furthermore, Cau et al. [56] discovered that MPs were present in the stomachs and intestines of 100 % of the total samples in two examined lobster species, the European spiny lobster (*Palinurus elephas*) and langoustine (*N*. *norvegicus*).

In summary, numerous species of crustaceans, such as crabs, shrimp, and lobsters, found globally are contaminated with MPs (Table 1). The size, type, abundance per individual, and absorption process of MPs vary across different species from various locations. The predominant MPs identified include fibers, fragments, and polyethylene, exhibiting a range of particle sizes from sub-micrometer to millimeter scales (Table 1). The absorption process typically takes place through ingestion or uptake via gills and digestive tracts (Table 1), underscoring the extensive prevalence and ecological implications of MP contamination in marine crustaceans. Multiple studies suggest that fibers are the predominant form of MPs found in crustaceans, with fragments, films, and beads following in prevalence [57,58]. The occurrence of fibers raises significant concerns, as their small size and extensive distribution in the marine environment foster easier ingestion [26]. Despite the growing evidence, there remain significant knowledge gaps regarding the bioaccumulation potential, retention times, and long-term physiological effects of MPs in decapods. Additional field and experimental investigations are essential to develop standardized detection techniques and to evaluate the consequences of MP contamination on seafood safety and public health.

**Table 1:** Evidence for the presence of microplastics (MPs) in various crustacean (crab, shrimp, and lobster) species and their size, type and absorption process.

Group	Scientific name	Location	Research type	Study part	MP size	MP abundance	MP type	Absorption process	Reference
Crab	*Gelasimus vocans*	Singapore's coastal habitats	Field	Hepatopancreas, digestive tract, and gills	730**–**1487 μm	(6.63 ± 0.97) items per individual	Polyethylene, polypropylene	Uptake	[123]
	*Austruca annulipes*	Singapore's coastal habitats	Field	Hepatopancreas, digestive tract, and gills	730**–**1487 μm	(12.18 ± 3.38) items per individual	Polyethylene, polypropylene	Uptake	[123]
	*Portunus sanguinolentus*	Gujarat, India	Field	Gut and gill	1**–**2 mm	(3.17 ± 2.46) items per individual	Polyethylene, polypropylene, polyethylene terephthalate, polyurethane, polystyrene	Uptake	[49]
	*Chiromantes dehaani*	Kokubu River Estuary, Japan	Field	Gut and gill	207**–**963 μm	(9.5 ± 6.6) items per individual	Polyolefins	Uptake	[124]
	*Chasmagnathus convexus*	Kokubu River Estuary, Japan	Field	Gut and gill	Median size to 1.6 mm	(26.2 ± 12.4) items per individual	Polyethylene	Uptake	[124]
	*Cardisoma armatum*	Ojo, Lagos, Nigeria	Field	Gills and stomachs	669 μm and 802.8 μm	**–**	Rayon, nylon	Uptake	[125]
	*Callinectes amnicola*	Ojo, Lagos, Nigeria	Field	Gills and stomachs	669 μm and 802.8 μm	**–**	Rayon, polyacrylate, nylon	Uptake	[125]
	*Neohelice granulata*	Bahía Blanca Estuary, Argentina	Field	Gills and digestive tract	Fibers: <500–1500 μm; fragment: <200 μm	**–**	Fiber	Uptake	[50]
	*Charybdis japonica*	Shengsi Islands, China	Field	Hepatopancreas	5 μm	Approximately 3 mg/g	Polystyrene microspheres	Filtration of water and consumption of mussels	[121]
	*Dorippe japonica*	Yellow Sea and East China Sea	Field	Gut, gills, and muscles	**–**	(5.88 ± 3.26) items per individual	Fibers, fragments, sheets, microbeads	Ingestion	[52]
	*Matuta planipes*	Yellow Sea and East China Sea	Field	Gills and guts	**–**	(5.17 ± 4.43) items per individual	Fibers, fragments, sheets, microbeads	Ingestion	[52]
	*Minuca rapax*	An estuary near Isla del Carmen, Campeche, Mexico	Field	Gill and hepatopancreas	53**–**63 μm	**–**	Polyethylene microspheres	Uptake	[126]
	*Charybdis bimaculata*	Zhoushan fishing ground, East China Sea	Field	Gastrointestinal tract	**–**	**–**	Fibers, fragments	**–**	[127]
	*Callinectis aminicola*	Pra Estuary, Ghana	Field	Soft tissues	<0.5 mm	(2.67 ± 1.44) items per individual	Polyethylene, polyester, polypropylene, polystyrene	Ingestion	[128]
	*Callinectes sapidus*	Corpus Christi Bay, Texas, the United States	Field	Stomach	**–**	0.87 items per individual	Fibers	Ingestion	[129]
	*Portunus trituberculatus*	Yellow Sea and East China Sea	Field	Gut, gills, and muscles	0–500 μm	(5.17 ± 4.43) items per individual	Fibers	Ingestion	[52]
	*Emerita analoga*	California coast, the United States	Field	Digestive tract	**–**	(0.65 ± 1.64) items per individual	Fibers	**–**	[130]
Shrimp	*Litopenaeus vannamei*	Sea area of the Republic of Korea	Field	Heads and intestines	Less than 100 μm	11.83 items/10 g	Polyethylene (55 %), polypropylene (31 %), polyethylene terephthalate (4 %), polystyrene (4 %)	Uptake	[131]
	*L*. *vannamei*	Hoai Nhon district, Binh Dinh Province, Vietnam	Field (cultured species)	Digestive tracts	302–3282 μm	(0.20 ± 0.12) items per individual	Fibers	Ingestion	[132]
	*Metapenaeus ensis*	Hoai Nhon district, Binh Dinh Province, Vietnam	Field	Digestive tracts	300**–**5000 μm	(2.26 ± 1.26) items per individual	Fibers	Ingestion	[132]
	*Penaeus semisulcatus*	Hoai Nhon district, Binh Dinh Province, Vietnam	Field	Digestive tracts	300**–**5000 μm	(1.03 ± 0.64) items per individual	Fibers	Ingestion	[132]
	*Penaeus monodon*	Hoai Nhon district, Binh Dinh Province, Vietnam	Field (cultured species)	Digestive tracts	300**–**2979 μm	(0.25 ± 0.12) items per individual	Fibers	Ingestion	[132]
	*P. monodon*	Negombo Lagoon, Sri Lanka	Field	Gastrointestinal tract and gills	>1000 μm	(4.72 ± 2.72) items per individual	Fibers, polystyrene, polyamide	Uptake	[133]
	*Penaeus indicus*	Negombo Lagoon, Sri Lanka	Field	Gastrointestinal tract and gills	>1000 μm	(3.13 ± 2.04) items per individual	Fibers, polystyrene, polyamide	Uptake	[133]
	*Metapenaeus monoceros*	Northern Bay of Bengal, Bangladesh	Field	Gastrointestinal tract	**–**	(3.87 ± 1.05) items per individual	Fibers	**–**	[47]
	*P*. *monodon*	Northern Bay of Bengal, Bangladesh	Field	Gastrointestinal tract	**–**	(3.40 ± 1.23) items per individual	Fibers	**–**	[47]
	*Penaeus notialis*	Pra estuary, Ghana	Field	Soft tissues	<0.5 mm	(1.64 ± 0.63) items per individual	Polyethylene, polyester	Ingestion	[128]
	*Fenneropenaeus indicus*	Cochin Estuary, India	Field	Soft tissues	500–1000 μm	(0.39 ± 0.6) items per individual	Polyacrylate, polyethylene, polystyrene, polypropylene	Ingestion	[134]
	*Parapenaeopsis hardwickii*	Xiangshan Bay, China	Field	Gastrointestinal tract, and muscle tissue	**–**	(0.25 ± 0.08) items per individual	Fibers	**–**	[135]
	*Palaemon varians*	Scandinavia to the Mediterranean	Laboratory	Digestive tract	0.1**–**9.9 μm	**–**	Fluorescent polystyrene microbeads	Ingestion	[136]
	*Crangon crangon*	Southern North Sea, Belgium	Field	Whole and peeled shrimp	200 μmup to 1000 μm	(1.23 ± 0.99) items per individual	Fibers	Ingestion	[137]
	*M*. *monoceros*	North East Arabian Sea	Field	Gastrointestinal tracts	100–250 μm	(7.23 ± 2.63) items per individual	Fibers	Ingestion	[138]
	*P*. *semisulcatus*	Musa Estuary, Persian Gulf	Field	Muscle and skin	<100 μm to >1000 μm	7.8 items per individual	Filamentous fragments	Ingestion	[25]
	*Penaeus occidentalis*	Tropical Eastern Pacific and Galápagos	Field	Digestive tracts and muscle tissue	**–**	**–**	Fragments	Ingestion	[139]
	*L*. *vannamei*	Malaysia and Ecuador	Field	Gastrointestinal tract	**–**	(21 ± 4) items/g in Malaysia, (13.4 ± 1.42) items/g in Ecuador	Film	–	[140]
Lobster	*Nephrops norvegicus*	North East Atlantic	Field	Gastrointestinal tract	**–**	(2.20 ± 2.47) items per individual	Polystyrene, polyamide (nylons), polypropylene	Ingestion	[141]
	*N*. *norvegicus*	West and northeast coasts of Ireland	Field	Gastrointestinal tract	0.143**–**16.976 mm	Approximately (1.75 ± 2.01) items per individual	Fibers, fragments, films	Ingestion	[57]

### 3.3 Trophic transfer of MPs

A major concern related to MP contamination in crustaceans is the possibility of trophic transfer within the food web [59]. Crustaceans serve as an important channel for the movement of MPs to various marine organisms. As filter-feeders and scavengers, these organisms can consume considerable amounts of MPs, which are subsequently transferred to their predators [60]. Decapods can gather pollutants, such as MPs, at levels significantly exceeding those present in their surroundings [61]. Research indicates that decapods such as *N*. *norvegicus* are capable of accumulating plastic in their stomachs [62]. This accumulation presents a possible risk to the health of various marine organisms that ingest them and, in the end, to human consumers. The occurrence of MPs in crustaceans underscores the complicated connections that exist between marine ecosystems and the wider implications of plastic pollution on the environment.

### 3.4 Effects on crustacean physiology and behavior

The ingestion of MPs by crustaceans can cause a range of physiological and behavioral effects that may seriously impair their survival and reproductive success. In the short term, MPs can reduce feeding efficiency, cause physical blockages in the digestive tract, and deplete energy reserves. When MPs accumulate in the digestive system, they obstruct the normal passage of food, leading to reduced food intake [5]. This decrease in nutrition limits the energy available for growth and reproduction, potentially resulting in stunted development, lowered fitness, and higher mortality rates over time.

Long-term physiological effects tend to be even more severe. Studies have linked MP ingestion to decreased reproductive success, including poorer egg quality, delayed larval development, and reduced egg retention [63]. For example, research on *Litopenaeus vannamei* showed that exposure to MPs and bisphenol A reduces the gonadosomatic index (GSI). The reduced GSI leads to a delay in the reproductive development of the gonads in shrimps [64]. Additionally, MPs can disrupt hormone levels and interfere with critical biological processes, further reducing reproductive outcomes [65].

MPs can induce oxidative stress in marine organisms, including bivalves, leading to increased activity of antioxidant enzymes like glutathione peroxidase and superoxide dismutase during short-term exposure [66]. These effects, especially in vital tissues such as the gills and liver, which are essential for respiration and metabolism, highlight the potential for significant physiological disruption in marine species. They can also weaken shrimp antiviral defenses, increasing susceptibility to pathogens like white spot syndrome virus [67]. Furthermore, MPs often carry heavy metals and persistent organic pollutants on their surfaces, intensifying toxicity through bioaccumulation. This results in liver damage, immune suppression, and greater vulnerability to illnesses [60].

Behavioral changes have also been observed, including altered feeding habits, decreased activity levels, and disrupted predator-prey interactions [68]. Given the crucial ecological role of crustaceans in maintaining marine ecosystem balance, these physiological and behavioral disruptions may have cascading effects throughout the ecosystem.

### 3.5 Human health risks from contaminated seafood

**Fig. 5:**
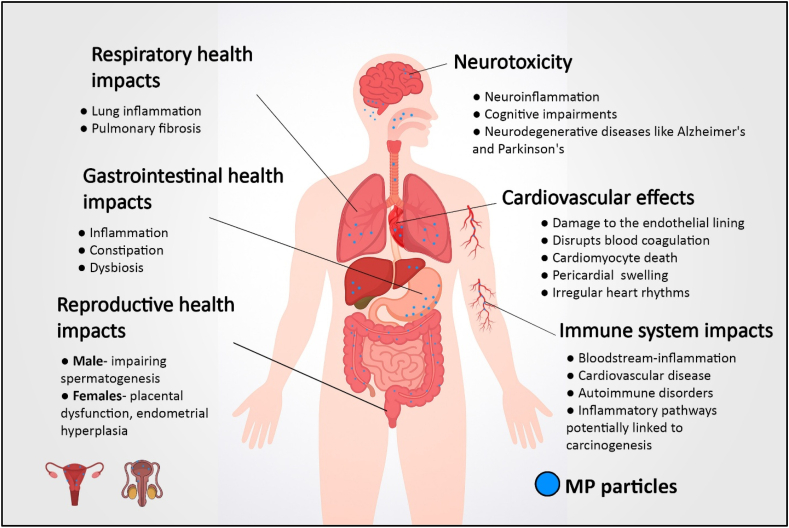
Potential health risks of microplastic (MP) contamination in seafood and their impact on human health.

Crustaceans play a crucial role in benthic-pelagic coupling and nutrient cycling, and they serve as food for higher trophic level organisms [69,70]. MPs found in crustaceans provide a direct pathway for human exposure, as these organisms are commonly consumed as seafood [71]. The presence of MPs can impact several human body systems, including the immune, respiratory, gastrointestinal, cardiovascular, neurological, and reproductive systems (Fig. 5).

#### 3.5.1 Impacts on immune system

MPs such as polystyrene can trigger immune system responses in the body [72,73]. When these particles enter the bloodstream or tissues, they may cause an inflammatory reaction [74]. Chronic inflammation has been linked to various health issues, including cardiovascular disease, autoimmune disorders, and cancer [[75], [76], [77]]. MPs can also accumulate in the lungs, leading to a surplus of immune cells and an increased risk of respiratory diseases [78,79]. Moreover, long-term exposure to MPs may contribute to the development of autoimmune conditions and elevate the risk of cancer [80,81].

#### 3.5.2 Impacts on respiratory health

MPs could be responsible for irritation and inflammation in the respiratory tract [82,83], leading to symptoms such as coughing, sneezing, wheezing, shortness of breath, and worsening asthma. Inhalation of MPs, particularly those from polystyrene or tire wear particles, can cause damage to the lungs. Breathing in airborne MPs also triggers lung inflammation and intensifies conditions like asthma or chronic obstructive pulmonary disease [84,85]. Furthermore, long-term exposure to inhaled MPs may result in pulmonary fibrosis, a serious condition that causes scar formation in the lung tissue [86,87].

#### 3.5.3 Impacts on gastrointestinal health

Exposure to MPs in the gastrointestinal tract has raised significant concerns due to their potential to cause inflammation, constipation, dysbiosis, and changes in intestinal absorption [88,89]. MPs can also accumulate in the digestive system, leading to irritation and gut blockages [90]. Additionally, these particles disrupt the symbiotic relationship between gut bacteria and their host, resulting in an imbalance known as dysbiosis [74]. This imbalance can weaken the host's immune system, potentially leading to chronic diseases, increased susceptibility to infections, and changes in genetic expression and gut microbiota composition [91,92].

#### 3.5.4 Cardiovascular effects

Exposure to MPs can lead to a range of health issues, including inflammation, oxidative stress, endothelial dysfunction, procoagulant activity, and cardiometabolic complications [93,94]. When MPs interact with the circulatory system, they can cause damage to the endothelial lining, disrupt blood coagulation, and interfere with metabolic processes, worsening cardiovascular health. These particles may contribute to cardiomyocyte death, pericardial swelling, and irregular heart rhythms by altering oxidative balance and causing inflammation [95,96]. The endothelial cells, which line the inner surfaces of blood vessels, are particularly vulnerable to MP exposure. Studies suggest that MPs may directly affect endothelial cells, causing cell death and disrupting vascular stability [97]. MPs can also increase the risk of thrombosis by promoting procoagulant activity [98,99]. Additionally, some studies suggest that MPs may indirectly influence cardiometabolic health by interfering with lipid metabolism, leading to dyslipidemia, a known risk factor for cardiovascular diseases [100,101].

#### 3.5.5 Neurotoxicity

Certain studies suggest that MPs, such as polystyrene, can affect the nervous system [102,103], potentially crossing the blood-brain barrier and causing neuroinflammation, which may lead to cognitive impairment [104]. Long-term exposure to MPs has also been linked to the development of neurodegenerative diseases, particularly Alzheimer's Suand Parkinson's diseases [105,106].

#### 3.5.6 Endocrine disruption

Endocrine disruption is recognized as a potential effect of MP exposure [74]. Some endocrine-disrupting compounds (EDCs) can interfere with normal hormonal processes, leading to harmful health effects [107]. Common EDCs, such as phthalate esters, bisphenol A, octylphenol, and nonylphenol, are byproducts or additives in plastics [108,109]. This disruption can negatively impact hormonal balance, reproductive health, development, and overall well-being [110,111].

#### 3.5.7 Cancer risk

MPs may elevate the risk of cancer by inducing chronic inflammation [74]. The ongoing inflammation triggered by the accumulation of MPs in tissues can amplify the likelihood of developing cancers, especially in the gastrointestinal tract and lungs [112]. Additionally, MPs can disrupt the microarchitecture of the lung, damaging tissue and facilitating the infiltration of cancer cells, which may promote tumor spread [[113], [114], [115]].

#### 3.5.8 Impacts on reproductive health

MPs, especially those embedded with EDCs, can negatively impact reproductive health by acting as physical and chemical stressors [116,117]. Once ingested or inhaled, MPs can enter systemic circulation and accumulate in reproductive tissues. Research indicates that MPs and their adsorbed toxicants like phthalates and bisphenol A disrupt the neuroendocrine system, interfering with hormone production via the hypothalamic-pituitary-gonadal axis [74]. In males, MPs may cross the blood-testis barrier by inducing oxidative stress and inflammatory responses, leading to impaired spermatogenesis and testicular damage [118]. In females, MPs have been shown to accumulate in the ovaries and uterus, where they can induce placental dysfunction, endometrial hyperplasia, ovarian atrophy, and fibrosis through hormone disruption and immune modulation [117].

All of the mentioned impacts pose significant public health risks, particularly for populations that rely heavily on seafood for protein intake. As such, the issue of MP contamination in seafood, particularly crustaceans, demands urgent attention to prevent further harm to human health.

### 3.6 Mitigation approaches to reduce MP pollution

Marine ecosystems are increasingly recognized as major sinks for MPs, with crustaceans being particularly vulnerable due to their ecological roles and feeding behaviors. In response, various mitigation strategies have been developed to lower MP pollution and its effects. A promising approach includes utilizing natural bioremediation systems, like filter-feeding organisms and seaweed, which have the capability to absorb or trap MPs from the water column. For example, species such as *Chaetomorpha linum* has demonstrated an ability to effectively capture MP particles in aquaculture environments, indicating their possible application in integrated multi-trophic aquaculture systems with crustaceans to reduce local MP concentrations [119]. Furthermore, the use of microbial degradation with strains like *Bacillus subtilis*, *Pseudomonas putida*, and *Rhodococcus sp.* presents a biological approach to break down synthetic polymers into less harmful components [11]. Microorganisms isolated from marine sediments have shown the ability to enzymatically degrade polyethylene and polystyrene under controlled conditions [120]. Technological methods like enhanced filtration systems in aquaculture effluent treatment have demonstrated effectiveness; the integration of ultrafiltration and advanced oxidation processes has achieved over 90 % efficiency in eliminating MPs from wastewater [121]. Minimising upstream sources is essential—this entails banning single-use plastics, creating biodegradable alternatives, and adopting sustainable product design to reduce the formation of secondary MPs. Ultimately, initiatives focusing on recycling that transform plastic waste into functional raw materials for aquaculture equipment (such as traps and nets) can minimize the introduction of new plastics into marine environments while fostering a circular economy [122]. The combination of these tailored mitigation strategies provides a viable route for reducing MP pollution and protecting crustacean populations within marine and coastal ecosystems.

### 3.7 Future directions for research

Although there is an increasing amount of literature regarding MP contamination in crustaceans, there are still considerable knowledge gaps that require additional exploration. Future investigations should emphasize the establishment of standard methodologies for sampling, quantifying, and identifying MPs across various crustacean species and habitats, supporting cross-study comparisons and meta-analyses. To comprehend the long-term impact of MP exposure on crustacean physiology, reproduction, immune function, and intergenerational impacts, longitudinal studies are essential. Furthermore, the majority of existing studies focus on ingestion within gastrointestinal tissues; however, subsequent research should explore translocation pathways and accumulation in edible tissues like muscles, which are directly associated with human consumption. A further interesting direction includes assessing the relationships between MPs and pathogens or toxicants in crustacean hosts, potentially revealing synergistic or antagonistic impacts on health and ecological fitness. Furthermore, it is essential to evaluate the consequences of MP pollution in aquaculture environments, particularly in areas with high crustacean farming, to guide effective environmental management and ensure seafood safety. Understanding species-specific differences in uptake, retention, and depuration capacity under variable environmental conditions will also help predict ecological risk. Ultimately, the integration of environmental monitoring with food safety assessments and public health surveillance can facilitate the creation of comprehensive policies designed to mitigate MP exposure in both ecosystems and human populations.

## 4 Conclusion

The presence of MP contamination in crustaceans is increasingly recognized as a significant environmental and public health issue. Crustaceans serve as essential elements within marine ecosystems and are a significant source of seafood. They face increased susceptibility to MP exposure through ingestion, sediment interaction, and trophic transfer. This review emphasizes the physiological, reproductive, and immunological effects of MPs on crustaceans, as well as the related human health risks associated with seafood consumption. Even with the growing focus in the scientific community, there are still considerable gaps in our comprehension of how different species accumulate substances, the long-term impacts, and the levels of contamination found in edible tissues. Combating MP pollution necessitates a comprehensive strategy that encompasses enhanced waste management, bioremediation techniques, technological advancements, and coordinated global regulatory measures. Future investigations should emphasize standard methodologies, molecular impact assessments, and monitoring frameworks that connect ecosystem and food safety. Protecting crustaceans against MP pollution is crucial for maintaining marine biodiversity and securing the safety of seafood supplies worldwide.

## CRediT authorship contribution statement

**Mohammad Shakil Khan:** Writing – review & editing, Writing – original draft, Methodology, Conceptualization. **Thowai Uching Marma:** Investigation, Data curation. **Samson Nahar Sumi:** Validation, Data curation. **Aniruddha Chisim:** Investigation, Data curation. **Ifthekher Ahmed Shakib:** Writing – review & editing. **Saifuddin Rana:** Writing – review & editing, Writing – original draft, Supervision, Conceptualization.

## Data availability

Data will be made available on request.

## Funding sources

This research did not receive any specific grant from funding agencies in the public, commercial, or not-for-profit sectors.

## Declaration of competing interest

The authors declare that they have no known competing financial interests or personal relationships that could have appeared to influence the work reported in this paper.
